# Prenatal famine exposure, adulthood obesity patterns and risk of type 2 diabetes

**DOI:** 10.1093/ije/dyx228

**Published:** 2017-11-17

**Authors:** Ruogu Meng, Jun Lv, Canqing Yu, Yu Guo, Zheng Bian, Ling Yang, Yiping Chen, Hui Zhang, Xiaofang Chen, Junshi Chen, Zhengming Chen, Lu Qi, Liming Li, the China Kadoorie Biobank Collaborative Group

**Affiliations:** 1 Peking University Health Science Center, Beijing, China; 2 Peking University Institute of Environmental Medicine, Beijing, China; 3 Chinese Academy of Medical Sciences, Beijing, China; 4 Nuffield Department of Population Health, University of Oxford, Oxford, UK; 5 Maiji Center for Disease Control and Prevention, Maiji, China; 6 Sichuan Center for Disease Control and Prevention, Chengdu, China; 7 China National Center for Food Safety Risk Assessment, Beijing, China; 8 School of Public Health and Tropical Medicine, Tulane University, New Orleans, LA, USA; 9 Harvard School of Public Health, Boston, MA, USA

**Keywords:** Starvation, obesity, type 2 diabetes

## Abstract

**Background:**

Prenatal exposure to famine and adulthood obesity have been independently related to the risk of type 2 diabetes; however, little is known about the joint effects of these risk factors at different stages of life on adulthood diabetes risk.

**Methods:**

The analysis included 88 830 participants of the China Kadoorie Biobank, who were born around the time of the Chinese Great Famine and without diabetes, cardiovascular diseases, or cancer at baseline. We defined famine exposure subgroups as nonexposed (born between 1 October 1962 and 30 September 964), fetal-exposed (born between 1 October 1959 and 30 September 1961) and early-childhood exposed (born between 1 October 1956 and 30 September 1958). General obesity was assessed by body mass index (BMI: overweight ≥ 24.0, obesity ≥ 28.0) and abdominal obesity assessed by waist-to-hip ratio (WHR, men/women: moderate ≥ 0.90/0.85, high ≥ 0.95/0.90).

**Results:**

During a median 7.3 years (642 552 person-years) of follow-up, we identified 1372 incident cases of type 2 diabetes. Compared with nonexposed and early-childhood exposed participants combined as a single comparison group, fetal-exposed participants showed an increased risk of diabetes in adulthood [hazard ratio (HR) = 1.25; 95% confidence interval (CI): 1.07–1.45]. The association between general obesity and diabetes was consistent across subgroups according to famine exposure (*P* for interaction > 0.05). A stronger association between abdominal obesity and diabetes was observed in the fetal-exposed subgroup than in other subgroups (*P* for interaction = 0.025 in the whole population). This interaction was more obvious in women (*P* = 0.013) but not in men (*P* = 0.699). Compared with normal-BMI and -WHR participants, those with both general (BMI ≥ 24.0) and abdominal (WHR ≥ 0.90/0.85) obesity in adulthood had 5.32 (95% CI: 3.81–7.43)-, 3.13 (2.48–3.94)- and 4.43 (3.45–5.68)-fold higher risks if these were carried during, before and after times of famine, respectively.

**Conclusions:**

Coexistence of prenatal experience of undernutrition and abdominal obesity in adulthood was associated with a higher risk of type 2 diabetes.

## Introduction

Since 1980, the diabetes burden has shown a faster increase in low- and middle-income countries than in high-income countries.^1^ The prevalence of diabetes has risen rapidly from 2.5% in 1994^2^ to 11.6% in 2010^3^ in China. The speedy increase of diabetes is parallel with the rise of obesity and the dramatic transition of traditional lifestyle toward unhealthy patterns.^4^ In addition, growing evidence has suggested that poor nutrition in early life also has profound effects on the predisposition to diabetes in later life.^5^^,^^6^

**Key Messages**

- The prenatal experience of the Chinese Great Famine was associated with an increased risk of adult type 2 diabetes, in this large prospective cohort study.
- Coexistence of prenatal famine experience and abdominal obesity in adulthood was associated with a higher risk of type 2 diabetes, especially in women.
- Participants who had childhood exposure to famine, and had both general and abdominal obesity in adulthood, had more than four times higher risks of diabetes than the non-obese.
- This study suggests that lifespan prevention may help relieve the growing burden of diabetes in China.

In epidemiological studies, low birthweight has been consistently related to later risk of type 2 diabetes.^7^ Analyses of populations with prenatal exposure to famine provide more a direct link between intrauterine malnutrition and adulthood risk of type 2 diabetes.^8–10^ A recent study found that the prevalence of hyperglycaemia associated with fetal exposure to famine was higher among individuals with higher adulthood body mass index (BMI), suggesting that prenatal exposure to famine may interact with obesity in later life to increase the risk of type 2 diabetes.^11^ In addition, recent studies suggest that distinct obesity patterns, such as combinations of general and abdominal obesity, may differently affect diabetes risk.^12^ However, no prospective study has assessed the joint associations of these factors with risk of type 2 diabetes.

The Chinese Great Famine of 1959–61 caused millions of excess deaths.^13^ It lasted over a relatively prolonged period in a previously chronically malnourished population who had not experienced significant nutritional transition until recent decades. In the present study, we prospectively examined whether early exposure to the Chinese Great Famine interacted with general and abdominal obesity in adulthood to increase the risk of incident type 2 diabetes in the China Kadoorie Biobank (CKB) cohort. In other words, we were interested in whether people who had ever experienced famine exposure in their early life would have a different risk of type 2 diabetes associated with the established risk factor of adult obesity.

## Methods

### Participants

As previously described,^14^^,^^15^ the CKB cohort was established during 2004–08 when 512 891 adults aged 30–79 years were enrolled from 10 study areas geographically spread across China, with valid baseline data including a completed questionnaire, physical measurements and a written informed consent form. The Ethical Review Committee of the Chinese Center for Disease Control and Prevention (Beijing, China) and the Oxford Tropical Research Ethics Committee, University of Oxford (UK), approved the study.

For the present study, we restricted the analysis to participants who were born between 1 October 1956 and 30 September 1964 (*n* = 120 492). The exact dates of the start and the end of the Chinese famine were not available and also not the same across regions. To minimize potential misclassification of the exposure periods, we excluded participants who were born between 1 October 1958 and 30 September 1959 (*n* = 11 579), and between 1October 1961 and 30 September 1962 (*n* = 14 861). Such exclusion was adopted in previous Chinese famine studies.^11^^,^^16^ We further excluded participants with previously diagnosed heart disease (*n* = 946), stroke (*n* = 545) or cancer (*n* = 313), and participants (n = 3598) who had a self-reported history of diabetes or screen-detected diabetes, defined as measured fasting blood glucose ≥7.0 mmol/l or random blood glucose ≥11.1 mmol/l at baseline. Such exclusion was conducted to prevent an incidence/prevalence bias and rule out the possibility of reverse causation for obesity patterns, adjusted lifestyle covariates and diabetes. Finally, the analysis included 88 830 participants.

### Assessment of famine exposure

All participants reported their date of birth at baseline. We categorized participants into three famine exposure subgroups: nonexposed (born between 1 October 1962 and 30 September 1964), fetal-exposed (born between 1 October 1959 and 30 September 1961) and early-childhood exposed (born between 1 October 1956 and 30 September 1958).

### Assessment of obesity patterns

At baseline, trained staff measured body weight, standing height, waist circumference and hip circumference, using calibrated instruments. BMI was calculated as weight in kilograms divided by height in metres squared. General obesity was determined by the BMI, categorized using the Chinese specific cutoff values as underweight (<18.5), normal (18.5–23.9), overweight (24.0–27.9) and obesity (>28.0).^17^ Waist-to-hip ratio (WHR) was calculated as waist circumference divided by hip circumference. Abdominal obesity was determined by the WHR, categorized as: normal (<0.90 for men and <0.85 for women), moderate (0.90–0.94 for men and 0.8–0.89 for women) and high (≥0.95 for men and ≥90 for women).^18^ Overweight/obesity (G, BMI ≥ 24.0) and abdominal obesity (A, WHR ≥ 0.90 for men and ≥0.85 for women) were also included in the analyses in the following joint categories: G+/A+ (both higher than normal), G-/A- (both normal), and G+/A- or G-/A+ (mixed).

### Assessment of covariates

Covariate information was obtained from baseline questionnaire including sociodemographic characteristics, lifestyle behaviuors, women’s reproductive information, and personal and family medical history. The daily level of physical activity was calculated by multiplying the metabolic equivalent tasks (METs) value for a particular type of physical activity by hours spent on that activity per day, and summing the MET-hours for all activities. Habitual dietary intake in the past year was assessed by a short qualitative food frequency questionnaire. Participants were considered as having a family history of diabetes if they reported at least one first-degree relative with diabetes. In a subsample of 1300 participants who completed the same questionnaire twice at an interval of <1.5 years, we observed moderate to excellent reproducibility for most of the lifestyle variables.^19^

### Ascertainment of type 2 diabetes

We identified incident diabetes since the participants’ enrolment into the study at baseline using linkage with local disease and death registries, with the recently established national health insurance system and by active follow-up.^14^ Trained staff, blinded to the baseline information, coded all cases with the 10th revision of the International Classification of Diseases (ICD-10). For the present analysis, we included diabetes cases coded as E11 and E14. Other cases clearly defined as non-type 2 diabetes were excluded. Because most participants in the present analysis were aged over 40 years, among whom the number of any non-type 2 diabetes was small, misclassification of other types of diabetes was unlikely. The validity of reported diabetes diagnosis has been adjudicated in a random sample of 831 cases by reviewing their medical records, of which 98.6% were confirmed.

### Statistical analysis

Analysis of variance were used to test differences in continuous variables, and binary logistic regressions were used to test the differences in categorical variables across subgroups according to famine exposure in early life, adjusting for age, sex and study areas as appropriate. We plotted the multivariable-adjusted proportion of joint categories of general and abdominal obesity according to famine exposure in early life, which was estimated using multinomial logistic regression. We also examined the association between famine exposure and adult obesity at baseline by using binary logistic regressions.

Participants contributed person-years from the date at baseline to the diagnosis of diabetes, death, loss to follow-up or 31 December 2013, whichever came first. A Cox proportional hazards regression model was used to estimate the hazard ratios (HRs) and 95% confidence intervals (CIs), with age as the underlying time scale and stratified by age at baseline in 5-year intervals and by study areas.

The multivariable model was adjusted for age (years), sex (for whole cohort only), education (no formal school, primary school, middle school, high school, collegeor university or higher), marital status (married, widowed, divorced or separated, or never married), smoking (never smoker, former smoker who quit for reasons other than illness, current smoker or former smoker who quit because of illness: 1–14, 15–24 or ≥25 cigarettes/day), alcohol consumption (non-weekly drinker, former weekly drinker, weekly drinker, daily drinker: <15, 15–29, 30–59 or ≥60 g/day), physical activity (MET-h/day), intakes of fruits, vegetables, red meat, white rice and wheat (day/calculated by assigning participants to the midpoint of their consumption category), family history of diabetes (yes or no) and menopausal status (premenopausal, perimenopausal or postmenopausal; for women only). For the analysis of the association of BMI with type 2 diabetes across different famine exposure subgroups, we further adjusted for WHR to examine the independent effect of BMI on type 2 diabetes. Similarly, BMI was additionally adjusted for the analysis of the association of WHR with type 2 diabetes. We also evaluated the association of the combination obesity patterns with the risk of type 2 diabetes. We tested the multiplicative interaction with sex or famine exposure by using a likelihood ratio test comparing models with and without the cross-product term. We assessed additive interaction by estimating the relative excess risk due to interaction (RERI). An RERI of 0 indicates no interaction on the additive scale and >0 indicates a synergistic interaction.^20^ We examined linear trend by modelling the median values for obesity measure categories as a continuous variable.

We calculated RERI using SAS (version 9.3, SAS Institute Inc., Cary, NC). All other statistical analyses were performed using Stata (version 13.1, 2013, StataCorp LP., College Station, TX).

## Results

### Early famine exposure and baseline obesity measures and covariates

**Figure 1:**
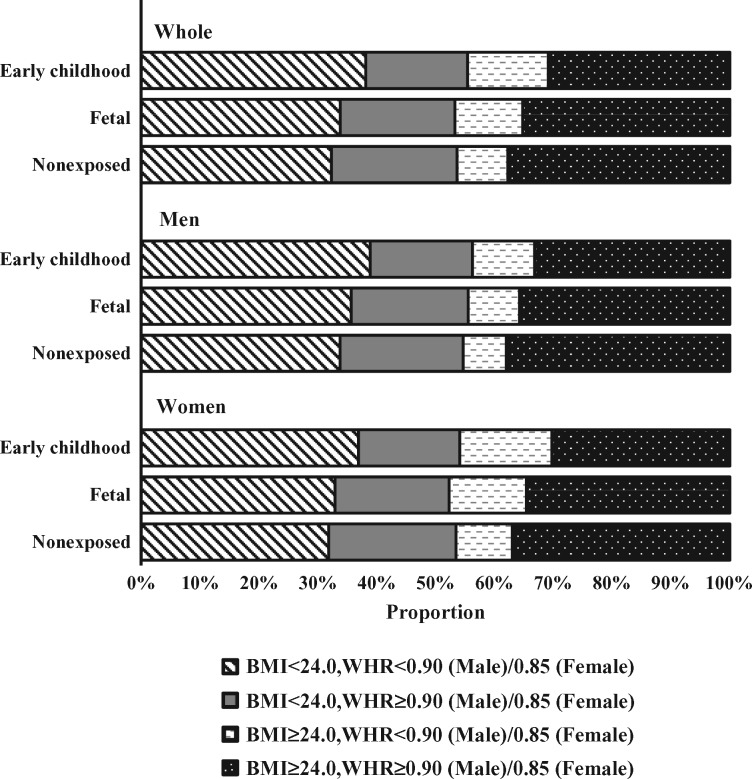
Multivariable-adjusted proportion of joint categories of general and abdominal obesity measures according to famine exposure in early life among 88 830 participants. BMI indicates body mass index; WHR, waist-to-hip ratio. Multivariable model was adjusted for age, sex, study area, education, marital status, smoking, alcohol consumption, physical activity, intakes of fruits, vegetables, red meat, white rice, and wheat, and menopausal status (for women only), as appropriate.

Table 1 presents the age-, sex-, and study area-adjusted baseline characteristics of 88 830 participants according to famine exposure in early life. Participants of younger age were more educated, less likely to smoke tobacco and drink alcohol, more physically active, less frequently to consume fruits and red meat and more frequently to consume wheat. Compared with nonexposed and early-childhood exposed participants, fetal-exposed participants were less likely to be rural residents. Multivariable-adjusted proportion showed that the three famine exposure subgroups had similar levels of general obesity (Figure 1). However, nonexposed and fetal-exposed participants had a higher prevalence of abdominal obesity than early-childhood participants, in both normal weight and overweight participants. To balance the differences in age between fetal-exposed and other two groups of participants, we combined both nonexposed and early-childhood exposed participants into one group. After adjustment for potential confounders, compared with this combined group of participants, famine exposure in the fetal stage was only associated with overweight/obesity in adult women (Supplementary Table 1, available as Supplementary data at *IJE* online).

**Table 1.** Baseline characteristics of 88 830 participants according to famine exposure in early life

	Nonexposed	Famine exposure	
		Fetal	Early childhood
No. of participants (%)	38588 (43.4)	18879 (21.3)	31363 (35.3)
Birth date			
From 1 Oct, year	1962	1959	1956
To 30 Sep, year	1964	1961	1958
Age at baseline, mean (SE), year	42.47 (0.01)	45.55 (0.01)	48.45 (0.01)
Male, no. (%)	14537 (37.7)	7336 (38.9)	12577 (40.1)
Rural area, no. (%)	21935 (56.8)	9572 (50.7)	17230 (54.9)
Middle school and above, no. (%)	29792 (77.5)	13465 (69.5)	17546 (56.6)
Married, no. (%)	36885 (94.6)	17827 (94.8)	29658 (95.4)
Family history of diabetes, no. (%)	3202 (8.4)	1834 (9.1)	2467 (8.1)
Postmenopausal women, no. (%)	1034 (14.9)	1370 (14.2)	6574 (17.7)
Daily smoking, no. (%)	9927 (25.7)	5162 (27.6)	8986 (28.4)
Weekly alcohol consumption, no. (%)	6092 (15.0)	3208 (17.0)	5337 (18.1)
Physical activity, mean (SE), MET-h/day	25.5 (0.1)	25.3 (0.1)	24.6 (0.1)
Average weekly consumption,^a^ mean (SE), day			
Fresh fruits	2.63 (0.02)	2.72 (0.02)	2.78 (0.02)
Fresh vegetables	6.81 (0.01)	6.86 (0.01)	6.91 (0.01)
Red meat	3.64 (0.02)	3.95 (0.02)	4.23 (0.02)
White rice	5.29 (0.01)	5.26 (0.01)	5.25 (0.01)
Wheat	3.91 (0.02)	3.79 (0.01)	3.60 (0.02)
BMI, mean (SE), kg/m^2^	23.81 (0.03)	23.90 (0.02)	23.83 (0.03)
WHR, mean (SE)	0.879 (0.001)	0.873 (0.000)	0.864 (0.001)

The results are presented as adjusted means or percentages, with adjustment for age, sex, and study area, as appropriate.

SE, standard error.

^a^Average weekly consumptions of fresh fruits, vegetables, red meat, white rice and wheat were calculated by assigning participants to the midpoint of their consumption category.

### Early famine exposure and type 2 diabetes

During a median 7.3 years (642 552 person-years) of follow-up, we identified 1372 incident type 2 diabetes. In the multivariable-adjusted analysis, participants exposed to famine in the fetal stage showed a slightly increased risk of diabetes in adulthood (Table 2). Compared with nonexposed participants, the multivariable-adjusted HRs (95% CIs) of type 2 diabetes were 1.21 (0.94–1.57) for fetal-exposed participants and 0.95 (0.67–1.36) for early-childhood exposed participants. The association of famine exposure with diabetes was similar between men and women (*P* = 0.835 for interaction with sex). When compared with nonexposed and early-childhood exposed participants combined, fetal-exposed participants showed increased risk of diabetes in adulthood [HRs (95% CI): 1.25 (1.07–1.45); *P* = 0.559 for interaction with sex).

**Table 2.** HRs (95% CIs) for incident type 2 diabetes according to famine exposure in early life among 88 830 participants

	Nonexposed	Famine exposure	
		Fetal	Early childhood
Whole cohort			
Cases	498	318	556
Case/PYs (1000)	1.78	2.34	2.45
Age-adjusted	1.00	1.20 (0.93–1.55)	0.91 (0.64–1.30)
Multivariable-adjusted^a^	1.00	1.19 (0.92–1.54)	0.90 (0.63–1.29)
Further adjusted for BMI and WHR^b^	1.00	1.21 (0.94–1.57)	0.95 (0.67–1.36)
Men			
Cases	183	121	186
Case/PYs (1000)	1.74	2.31	2.06
Age-adjusted	1.00	1.25 (0.82–1.91)	0.77 (0.43–1.39)
Multivariable-adjusted^a^	1.00	1.26 (0.82–1.93)	0.81 (0.45–1.48)
Further adjusted for BMI and WHR^b^	1.00	1.27 (0.83–1.94)	0.83 (0.46–1.51)
Women			
Cases	315	197	370
Case/PYs (1000)	1.80	2.36	2.72
Age-adjusted	1.00	1.16 (0.84–1.60)	0.96 (0.62–1.49)
Multivariable-adjusted^a^	1.00	1.14 (0.83–1.58)	0.93 (0.59–1.45)
Further adjusted for BMI and WHR^b^	1.00	1.15 (0.83–1.60)	0.98 (0.62–1.54)

PYs, person-years.

^a^Multivariable model was adjusted for age (years), sex (for whole cohort only), education (no formal school, primary school, middle school, high school, college, or university or higher), marital status (married, widowed, divorced or separated, or never married), smoking (never smoker, former smoker who quit for reasons other than illness, current smoker or former smoker who quit because of illness: 1–14, 15–24 o ≥25 cigarettes/day), alcohol consumption (non-weekly drinker, former weekly drinker, weekly drinker, daily drinker: <15, 15–29, 30–59 or ≥60 g/day), physical activity (MET-h/day), intakes of fruits, vegetables, red meat, white rice and wheat (day/week; calculated by assigning participants to the midpoint of their consumption category), family history of diabetes (yes or no), and menopausal status (premenopausal, perimenopausal or postmenopausal; for women only).

^b^Further adjusted for BMI (<18.5, 18.5–23.9, 24.0–27.9 or ≥28.0) and WHR (men: <0.90, 0.90–0.94 or ≥0.95; women: <0.85, 0.85–0.89 or ≥0.90).

### Early famine exposure, obesity measures and type 2 diabetes

After mutual adjustment for both obesity measures and other covariates, both overweight/obesity and abdominal obesity were independently associated with an increased risk of type 2 diabetes in the whole cohort, men, and women (all *P* < 0.05 for linear trend) (Table 3). The association between BMI and diabetes was consistent across subgroups according to famine exposure (*P* for interaction with famine exposure > 0.05). We observed a stronger association between abdominal obesity and diabetes in the fetal-exposed subgroup than in other subgroups (*P* for interaction with famine exposure = 0.025). After adjustment for BMI and other covariates, the HRs (95% CIs) of type 2 diabetes in the fetal-exposed subgroup were 1.98 (1.43–2.74) and 3.03 (2.17–4.24) for moderate and high of abdominal obesity, respectively, which were higher than those in the nonexposed [HRs (95% CIs): 1.54 (1.20–1.99) and 2.41 (1.85–3.13)] and early-childhood exposed [HRs (95% CIs): 1.30 (1.03–1.65) and 1.85 (1.45–2.36)] participants. This trend was similarly observed in both men and women, but appeared to be more obvious in women (*P* for interaction: 0.699 in men and 0.013 in women). When comparing the fetal-exposed subgroup with the other two subgroups combined, we observed positive additive interactions of fetal famine exposure with abdominal obesity in the whole cohort and women, with all corresponding RERI > 0 (Supplementary Table 2, available as Supplementary data at *IJE* online).

**Table 3.** Multivariable-adjusted HRs (95% CIs) for association between adult obesity measures and type 2 diabetes according to famine exposure in early life among 88 830 participants

	Cases	Case/PYs (1000)	Nonexposed	Famine exposure		*P*_interaction_
				Fetal	Early childhood	
BMI at baseline,^a^ kg/m^2^						
Whole cohort						
<24.0	431	1.23	1.00	1.00	1.00	0.225
24.0–27.9	605	2.68	1.78 (1.42–2.24)	1.83 (1.38–2.44)	1.57 (1.27–1.94)	
≥28.0	336	5.11	3.32 (2.53–4.38)	2.92 (2.06–4.14)	2.93 (2.26–3.81)	
Men						
<24.0	142	1.03	1.00	1.00	1.00	0.859
24.0–27.9	220	2.54	1.74 (1.16–2.61)	1.94 (1.18–3.18)	1.99 (1.34–2.94)	
≥28.0	128	5.47	3.94 (2.44–6.36)	3.49 (1.91–6.38)	4.26 (2.61–6.95)	
Women						
<24.0	289	1.36	1.00	1.00	1.00	0.077
24.0–27.9	385	2.77	1.84 (1.40–2.42)	1.79 (1.26–2.54)	1.40 (1.09–1.81)	
≥28.0	208	4.92	3.01 (2.14–4.24)	2.63 (1.70–4.05)	2.47 (1.81–3.38)	
WHR at baseline^b^						
Whole cohort						
Men < 0.90, women < 0.85	351	1.18	1.00	1.00	1.00	0.025
Men 0.90–0.94, women 0.85–0.89	409	2.17	1.54 (1.20–1.99)	1.98 (1.43–2.74)	1.30 (1.03–1.65)	
Men ≥ 0.95, women ≥ 0.90	612	3.88	2.41 (1.85–3.13)	3.03 (2.17–4.24)	1.85 (1.45–2.36)	
Men						
<0.90	110	0.98	1.00	1.00	1.00	0.699
0.90–0.94	140	1.89	1.47 (0.95–2.28)	1.77 (1.00–3.12)	1.54 (1.00–2.39)	
≥0.95	240	3.88	2.05 (1.29–3.25)	3.17 (1.80–5.60)	2.22 (1.41–3.47)	
Women						
<0.85	241	1.31	1.00	1.00	1.00	0.013
0.85–0.89	269	2.36	1.57 (1.15–2.15)	2.17 (1.45–3.23)	1.25 (0.94–1.65)	
≥0.90	372	3.88	2.73 (1.97–3.77)	3.05 (1.99–4.68)	1.74 (1.30–2.34)	

PYs, person-years.

Multivariable model was adjusted for age (years), sex (for whole cohort only), education (no formal school, primary school, middle school, high school, college, or university or higher), marital status (married, widowed, divorced or separated, or never married), smoking (never smoker, former smoker who quit for reasons other than illness, current smoker or former smoker who quit because of illness: 1–14, 15–24 or ≥25 cigarettes/day), alcohol consumption (non-weekly drinker, former weekly drinker, weekly drinker, daily drinker: <15, 15–29, 30–59 or ≥60 g/day), physical activity (MET-h/day), intakes of fruits, vegetables, red meat, white rice and wheat (day/week; calculated by assigning participants to the midpoint of their consumption category), family history of diabetes (yes or no) and menopausal status (premenopausal, perimenopausal or postmenopausal; for women only).

^a^Analysis of BMI was further adjusted for WHR (men: <0.90, 0.90–0.94 or ≥0.95; women: <0.85, 0.85–0.89 or ≥0.90).

^b^Analysis of WHR was further adjusted for BMI (<18.5, 18.5–23.9, 24.0–27.9 or ≥28.0).

We further analysed the relative risks for the joint categories of overweight/obesity and abdominal obesity with type 2 diabetes according to famine exposure, with the category of both lower BMI and lower WHR as the reference (Table 4). Compared with the reference group, participants with both overweight/obesity and abdominal obesity had the higher risks of developing type 2 diabetes in the fetal-exposed subgroup than in the nonexposed and early-childhood exposed subgroups (*P* = 0.047 for interaction with famine exposure); the respective multivariable-adjusted HRs (95% CIs) were 5.32 (3.81–7.43), 4.43 (3.45–5.68) and 3.13 (2.48–3.94) in the whole cohort. The interaction between the joint categories of obesity and famine exposure was clearly observed in women (*P* for interaction = 0.036), but not in men (*P* for interaction = 0.923).

**Table 4.** Multivariable-adjusted HRs (95% CIs) for joint association of general and abdominal obesity with risk of incident type 2 diabetes according to famine exposure in early life among 88 830 participants

**Abdominal obesity****^a^**	**Overweight/ obesity**^b^	**Cases**	**Case/PYs (1000)**	**Nonexposed**	**Famine exposure**		***P*_interaction_**
					**Fetal**	**Early childhood**	
Whole cohort							
No	No	220	0.97	1.00	1.00	1.00	0.047
	Yes	131	1.87	2.17 (1.51–3.11)	1.80 (1.10–2.95)	2.04 (1.47–2.85)	
Yes	No	211	1.69	1.91 (1.39–2.63)	2.19 (1.44–3.33)	1.64 (1.23–2.19)	
	Yes	810	3.66	4.43 (3.45–5.68)	5.32 (3.81–7.43)	3.13 (2.48–3.94)	
Men							
No	No	81	0.90	1.00	1.00	1.00	0.923
	Yes	29	1.33	1.61 (0.80–3.25)	1.51 (0.59–3.89)	1.97 (1.01–3.85)	
Yes	No	61	1.28	1.47 (0.85–2.56)	1.87 (0.93–3.77)	1.61 (0.95–2.74)	
	Yes	319	3.61	3.89 (2.58–5.85)	5.69 (3.32–9.74)	4.52 (3.03–6.73)	
Women							
No	No	139	1.02	1.00	1.00	1.00	0.036
	Yes	102	2.12	2.47 (1.61–3.79)	1.92 (1.07–3.46)	2.01 (1.37–2.96)	
Yes	No	150	1.95	2.22 (1.50–3.28)	2.45 (1.44–4.17)	1.69 (1.19–2.39)	
	Yes	491	3.69	4.82 (3.50–6.64)	5.32 (3.45–8.22)	2.67 (2.00–3.57)	

PYs, person-years.

Multivariable model was adjusted for age (years), sex (for whole cohort only), education (no formal school, primary school, middle school, high school, college, or university or higher), marital status (married, widowed, divorced or separated, or never married), smoking (never smoker, former smoker who quit for reasons other than illness, current smoker or former smoker who quit because of illness: 1–14, 15–24 or ≥25 cigarettes/day), alcohol consumption (non-weekly drinker, former weekly drinker, weekly drinker, daily drinker: <15, 15–29, 30–59 or ≥60 g/day), physical activity (MET-h/day), intakes of fruits, vegetables, red meat, white rice and wheat (day/week; calculated by assigning participants to the midpoint of their consumption category), family history of diabetes (yes or no), and menopausal status (premenopausal, perimenopausal or postmenopausal; for women only).

**^a^**WHR: men ≥ 0.90, women ≥ 0.85.

^b^BMI ≥ 24.0 kg/m^2^.

## Discussion

In this large prospective cohort of over 88 000 adults born around the time of the Chinese Great Famine, famine exposure in the fetal stage was associated with an increased risk of type 2 diabetes in adulthood. The association between adulthood abdominal obesity and type 2 diabetes was stronger in participants with fetal exposure to famine than in other groups. Compared with nonobese participants defined by both BMI and WHR, those with famine exposure and with both general and abdominal obesity in adulthood had more than four times higher risk of type 2 diabetes.

Previous Chinese studies have shown that early life exposure to famine was associated with an increased risk of type 2 diabetes in adulthood.^11^^,^^21–26^ Li *et al.* suggested that most effects commonly attributed to the Chinese famine of 1959–61 could be explained by uncontrolled age differences between famine births and control births.^27^ When they compared famine births with pre- and post-famine births combined as a single control group to control for the age effect in the meta-analysis, no famine effect was seen for type 2 diabetes (OR = 0.96; 95% CI: 0.73–1.28). In the present prospective study, we were less concerned with selective survival bias, that previous cross-sectional studies might suffer from, and corrected for the age imbalance by combining pre- and post-famine births. We still observed an increased risk of type 2 diabetes in fetal-exposed participants. In addition, the association between fetal famine exposure and diabetes in adulthood did not change materially after adjustment for BMI and WHR, suggesting that adult obesity might not be a mediator of the association.

Our findings, consistent with previous studies,^10^^,^^11^ indicated that early famine exposure did not interact with general obesity during adulthood to influence diabetes risk. However, our study for the first time found that the experience of poor nutrition in prenatal life significantly exacerbated the effect of abdominal obesity on the risk of type 2 diabetes. Accumulating evidence suggests that maternal undernutrition may result in permanent changes in the function and mass of pancreatic β-cells and the sensitivity of tissues to insulin.^28^ Abdominal obesity in adulthood has been linked to insulin resistance through increased release of free fatty acids and abnormal secretion of adipokines.^12^ As compared with Caucasians, Asians have relatively higher prevalence of abdominal obesity, which may partly account for the different risk of type 2 diabetes in these populations.^29^ Our findings are in line with the thrifty phenotype hypothesis that the emergence of pathological changes following undernutrition in early life may be dependent upon the superimposition of later life risk factors such as abdominal obesity.^6^

In the present study, we found that the exacerbation by exposure to famine in early life of the association between abdominal obesity and type 2 diabetes was more apparent in women than in men. Several possible mechanisms might explain such gender difference. For example, male and female fetuses adapt differently to developmental challenges; and sex steroids have a profound influence on the development and progression of developmentally programmed disease states.^30^ A study of Dutch famine-exposed participants indicated that an adverse prenatal environment triggered persistent changes in DNA methylation and that these changes differed by sex.^31^ In addition, visceral adipose tissue has shown a stronger relation with the risk of type 2 diabetes in adult women than in men, suggesting that visceral fat accumulation may be particularly detrimental among women.^32^ Further studies are needed to investigate the underlying mechanisms by which biological sex contributes to the difference.

To the best of our knowledge, this study is the first to jointly investigate the relations of early life exposure to famine and adulthood obesity patterns with the risk of type 2 diabetes. The strengths of this study include a large sample size and careful control for potential confounding factors. The inclusion of a geographically diverse population living in urban and rural areas makes our results generalizable to the Chinese. The prospective design, with the exclusion of prevalent cases of diabetes at baseline, could prevent incidence-prevalence bias and minimize the possibility of reverse causation for adiposity measures, lifestyle covariates and diabetes. The anthropometric information was measured rather than self-reported, providing more accurate estimates of general and abdominal obesity.

Our study has several limitations. Individual data for famine exposure were absent. The specific period and severity of the Chinese Great Famine varied across regions. Because of a lack of officially well-documented famine severity across sub-provincial regions of China, we also did not considered participants’ birthplace in the definition of famine exposure. Misclassification of famine exposure was inevitable, but should be nondifferential on subsequent disease status and should have biased our results toward the null. The definition of famine exposure in the present study was consistent with previous studies, with the exclusion of participants born in 1 year between three famine exposure subgroups, to minimize potential misclassification.^11^^,^^16^ Lack of information on exposures and medical conditions in early life might lead to residual confounding. In the present study, the questionnaire on lifestyle covariates has not yet been validated directly. It was. however, was adapted from validated questionnaires used in several other studies, with some additional modifications after a pilot study. Such measurement errors may also result in residual confounding. In addition, the ascertainment of incident diabetes in the CKB cohort relied mainly on the health insurance system; and under-detection of asymptomatic diabetes was likely to be non-differential and might lead to attenuation of effect estimates.

In conclusion, we found that the coexistence of experience of poor nutrition in prenatal life and abdominal obesity in adulthood was associated with a higher risk of type 2 diabetes. This finding may at least partly explain the emerging epidemic of type 2 diabetes in the contemporary Chinese adults, who experienced chronic undernutrition in early life and are troubled with obesity, especially abdominal obesity, in later life. Our study lends support to the preventive measures across the lifespan, from prenatal to later life, to mitigate diabetes risk in Chinese.

## Supplementary Data

Supplementary data are available at IJE online.

## Funding

This work was supported by grants (81373082, 81390544) from the National Natural Science Foundation of China. The CKB baseline survey and the first re-survey were supported by a grant from the Kadoorie Charitable Foundation in Hong Kong. The long-term follow-up is supported by grants from the UK Wellcome Trust (088158/Z/09/Z, 104085/Z/14/Z) and a grant from the Chinese Ministry of Science and Technology (2011BAI09B01). J.L. is supported by the State Scholarship Fund of China Scholarship Council (201506015053). L.Q. is supported by NIH grants from the National Heart, Lung, and Blood Institute (HL071981, HL034594, HL126024, HL132254), the National Institute of Diabetes and Digestive and Kidney Diseases (DK091718, DK100383, DK078616), the Boston Obesity Nutrition Research Center (DK46200) and a United States – Israel Binational Science Foundation Grant (2011036). L.Q. was a recipient of the American Heart Association Scientist Development Award (0730094N). The funders had no role in the study design, data collection, data analysis and interpretation, writing of the report or the decision to submit the article for publication.

## Supplementary Material

Supplementary Data
