# Supplementary material for: Prenatal famine exposure, adulthood obesity patterns and risk of type 2 diabetes
Source: Int J Epidemiol. 2017 Nov 17;47(2):399–408. doi: 10.1093/ije/dyx228 (PMC5913613; doi:10.1093/ije/dyx228)
Supplement: Supplementary Data [file dyx228_ije-2017-05-0552-file003.docx]

**Supplementary Table 1. ORs (95% CIs) for adult obesity according to famine exposure in early life among 88 830 participants**

|  | **Nonexposed and**  **early-childhood exposed** | **Fetal exposed** | **P _interaction with sex_** |
| --- | --- | --- | --- |
| **Overweight/obesity (BMI ≥24.0 kg/m^2^)** | | | 0.019 |
| **Whole cohort** |  |  |  |
| Cases | 31,631 | 8,807 |  |
| Multivariable-adjusted | 1.00 | 1.04 (1.01, 1.08) |  |
| **Men** |  |  |  |
| Cases | 11,988 | 3,321 |  |
| Multivariable-adjusted | 1.00 | 0.96 (0.90, 1.03) |  |
| **Women** |  |  |  |
| Cases | 19,643 | 5,486 |  |
| Multivariable-adjusted | 1.00 | 1.07 (1.02, 1.12) |  |
| **Abdominal obesity (WHR: ≥0.90 in men and ≥0.85 in women)** | | | <0.001 |
| **Whole cohort** |  |  |  |
| Cases | 38,230 | 10,115 |  |
| Multivariable-adjusted | 1.00 | 1.02 (0.98, 1.06) |  |
| **Men** |  |  |  |
| Cases | 14,944 | 4,072 |  |
| Multivariable-adjusted | 1.00 | 1.05 (0.99, 1.12) |  |
| **Women** |  |  |  |
| Cases | 23,286 | 6,043 |  |
| Multivariable-adjusted | 1.00 | 1.00 (0.96, 1.05) |  |

Abbreviations: OR indicates odds ratio; CI, confidence interval; BMI, body mass index; WHR, waist-to-hip ratio; and MET, metabolic equivalent of task.

Multivariable model was adjusted for age (years), sex (for whole cohort only), study area, education (no formal school, primary school, middle school, high school, college, or university or higher), marital status (married, widowed, divorced or separated, or never married), smoking (never smoker, former smoker who have quit for reasons other than illness, current smoker or former smoker who have quit because of illness: 1-14, 15-24, or ≥25 cigarettes/day), alcohol consumption (non-weekly drinker, former weekly drinker, weekly drinker, daily drinker: <15, 15-29, 30-59, or ≥60 g/day), physical activity (MET-hour/day), intakes of fruits, vegetables, red meat, white rice, and wheat (day/week; calculated by assigning participants to the midpoint of their consumption category), and menopausal status (premenopausal, perimenopausal, or postmenopausal; for women only). Analysis of BMI was further adjusted for WHR (Men: <0.90, 0.90-0.94, or ≥0.95; women: <0.85, 0.85-0.89, or ≥0.90). Analysis of WHR was further adjusted for BMI (<18.5, 18.5-23.9, 24.0-27.9, or ≥28.0).

**Supplementary Table 2. Additive interaction analysis of fetal famine exposure and adult obesity on type 2 diabetes among 88 830 participants**

| **Fetal famine exposure** | **Adult obesity** | **No. of cases** | **Cases/PYs (1000)** | **HR (95% CI)** | **P^*^** | **RERI** |
| --- | --- | --- | --- | --- | --- | --- |
| **Overweight/obesity (BMI≥24.0kg/m^2^)** | | | | | | |
| **Whole cohort** | | | | | | |
| No | No | 342 | 1.2 | 1.00 | 0.588 | 0.01 (-0.03, 0.05) |
| No | Yes | 712 | 3.1 | 1.85 (1.60, 2.13) |  |  |
| Yes | No | 89 | 1.2 | 1.10 (0.86, 1.41) |  |  |
| Yes | Yes | 229 | 3.6 | 2.45 (2.01, 2.98) |  |  |
| **Men** | | | | | | |
| No | No | 110 | 1.0 | 1.00 | 0.503 | -0.02 (-0.09, 0.04) |
| No | Yes | 259 | 3.0 | 2.08 (1.61, 2.69) |  |  |
| Yes | No | 32 | 1.1 | 1.30 (0.85, 1.98) |  |  |
| Yes | Yes | 89 | 3.8 | 3.02 (2.14, 4.27) |  |  |
| **Women** | | | | | | |
| No | No | 232 | 1.4 | 1.00 | 0.137 | 0.04 (-0.01, 0.08) |
| No | Yes | 453 | 3.2 | 1.76 (1.48, 2.10) |  |  |
| Yes | No | 57 | 1.3 | 1.03 (0.76, 1.40) |  |  |
| Yes | Yes | 140 | 3.6 | 2.21 (1.73, 2.82) |  |  |
| **Abdominal obesity (WHR≥0.90 in men, ≥0.85 in women)** | | | | | | |
| **Whole cohort** | | | | | | |
| No | No | 282 | 1.2 | 1.00 | <0.001 | 0.13 (0.09, 0.17) |
| No | Yes | 772 | 2.8 | 1.66 (1.42, 1.93) |  |  |
| Yes | No | 69 | 1.1 | 0.95 (0.72, 1.26) |  |  |
| Yes | Yes | 249 | 3.5 | 2.26 (1.85, 2.77) |  |  |
| **Men** | | | | | | |
| No | No | 87 | 1.0 | 1.00 | 0.185 | 0.05 (-0.02, 0.11) |
| No | Yes | 282 | 2.6 | 1.75 (1.33, 2.30) |  |  |
| Yes | No | 23 | 1.0 | 1.12 (0.69, 1.81) |  |  |
| Yes | Yes | 98 | 3.4 | 2.63 (1.85, 3.74) |  |  |
| **Women** | | | | | | |
| No | No | 195 | 1.4 | 1.00 | <0.001 | 0.18 (0.13, 0.23) |
| No | Yes | 490 | 2.9 | 1.66 (1.38, 2.00) |  |  |
| Yes | No | 46 | 1.1 | 0.92 (0.66, 1.29) |  |  |
| Yes | Yes | 151 | 0.9 | 2.13 (1.66, 2.73) |  |  |

Abbreviations: HR indicates hazard ratio; CI, confidence interval; PYs, person-years; RERI, relative excess risk due to interaction; MET indicates metabolic equivalent of task; BMI, body mass index; and WHR, waist-to-hip ratio.

Multivariable model was adjusted for age (years), sex (for whole cohort only), education (no formal school, primary school, middle school, high school, college, or university or higher), marital status (married, widowed, divorced or separated, or never married), smoking (never smoker, former smoker who have quit for reasons other than illness, current smoker or former smoker who have quit because of illness: 1-14, 15-24, or ≥25 cigarettes/day), alcohol consumption (non-weekly drinker, former weekly drinker, weekly drinker, daily drinker: <15, 15-29, 30-59, or ≥60 g/day), physical activity (MET-hour/day), intakes of fruits, vegetables, red meat, white rice, and wheat (day/week; calculated by assigning participants to the midpoint of their consumption category), family history of diabetes (yes or no), and menopausal status (premenopausal, perimenopausal, or postmenopausal; for women only). Analysis of overweight/obesity was further adjusted for WHR (Men: <0.90, 0.90-0.94, or ≥0.95; women: <0.85, 0.85-0.89, or ≥0.90). Analysis of abdominal obesity was further adjusted for BMI (<18.5, 18.5-23.9, 24.0-27.9, or ≥28.0).

^*^P for interaction on an additive scale.
